# ‘It causes me to minimise myself’: impostor phenomenon in simulation educators

**DOI:** 10.1186/s41077-025-00369-9

**Published:** 2025-07-22

**Authors:** Kirsty J. Freeman, Debra Nestel, Stephen Houghton, Sandra E. Carr

**Affiliations:** 1 Rural Clinical School of Western Australia, The University of Western Australia, Crawley, Perth, WA 6009 Australia; 2 Simulation Education in Healthcare, Monash University, Melbourne, Australia; 3 Surgical Education, Department of Surgery, University of Melbourne, Melbourne, Australia; 4 Graduate School of Education at the University of Western Australia, Perth, Australia; 5 Health Professions Education at the University of Western Australia, Melbourne, Australia

**Keywords:** Impostor phenomenon, Healthcare simulation educators, Faculty development, Hermeneutic phenomenology

## Abstract

**Background:**

Impostor phenomenon (IP) is a common experience among healthcare professionals, characterised by persistent feelings of inadequacy, fear of being exposed as a fraud, and self-doubt, despite external evidence of competence. In healthcare simulation, where educators frequently transition between roles and responsibilities, little is known about how simulation educators experience and navigate IP throughout their careers. This study aims to explore the lived experiences of IP among healthcare simulation educators.

**Methods:**

The study builds on our earlier work in which simulation educators used the Clance Impostor Phenomenon Scale for identifying self-reported IP. Participants were recruited through professional networks. Using a hermeneutic phenomenological approach, we explored the lived experiences of 20 simulation educators. Semi-structured interviews were conducted, audio-recorded, and transcribed verbatim. Data were analysed using an iterative process of interpretation grounded in hermeneutic inquiry.

**Results:**

Four themes were identified: (1) *I don’t have the right badges*, where educators described feeling unqualified and in constant need of external validation; (2) *Now you see me, now you don’t*, illustrating how IP led participants to minimise themselves in professional settings; (3) *Friend or foe*, revealing the dual role of IP as both a motivator and a source of insecurity; and (4) *Hello, my old friend*, highlighting the cyclical nature of IP, where feelings of self-doubt resurface.

**Conclusions:**

IP is a persistent and cyclical experience among healthcare simulation educators. While IP can drive some educators to strive for excellence, it can also lead to anxiety, self-minimisation, and missed opportunities. We call on the healthcare simulation community to develop and study strategies such as tailored professional development, mentorship, and communities of practice, to support educators in managing and mitigating negative impacts of IP on performance and well-being.

## Background

In a recent international study of simulation educators, approximately half reported experiencing feelings of IP [1]. First described by Clance and Imes in 1978, IP is the internal experience of believing that oneself is not as competent as others perceive them to be, despite evidence of accomplishments [2]. While their work focused on women, subsequent research has shown that IP affects individuals across a wide range of professions, gender identities, and cultural backgrounds [3–5].

Although the term ‘impostor syndrome’ often appears in the literature as an alternative name, our research team, and others publishing more recently, prefer the term ‘impostor phenomenon.’

This term aligns with existing literature, which distinguishes *impostor phenomenon* as a common, situation or contextual experience rather than a pathological condition [6]. The distinction is significant: ‘syndrome’ typically refers to a cluster of symptoms associated with a medical or psychological disorder, whereas ‘phenomenon’ describes an observable experience shaped by social and environmental contexts.

Individuals experiencing IP report intense feelings of self-doubt, anxiety, and fear, often feeling undeserving of their accomplishments [2, 6, 7]. IP is not thought to be something educators outgrow with career progression; instead, it happens cyclically, triggered by new jobs, projects, or responsibilities [2, 6]. Repeated experiences negatively impact well-being, job satisfaction, and career progression [8, 9].

IP is widely experienced among healthcare professionals at all career stages, from students to experienced physicians, correlating with burnout, stress, and reduced job satisfaction [10–12].

Understanding how simulation educators experience IP is crucial as they frequently transition between clinical and educational roles. These transitions may lead to vulnerabilities, especially where they may be more experienced in their clinical rather than their educator role. Insights into how IP manifests and impacts this population could inform targeted support strategies, ultimately strengthening the simulation education workforce. Despite growing awareness of the prevalence of IP in individuals within the simulation education community [1], we do not know what it means to navigate lived experience of IP. This study aimed to explore simulation educators lived experiences of IP, to better understand how the phenomenon manifests and impacts them and their careers.

## Methods

### Study design

This study adopted a hermeneutic phenomenological approach, specifically drawing on the work of van Manen, to explore the lived experiences of healthcare simulation educators experiencing IP [13]. Hermeneutic phenomenology focuses on the interpretation of lived experiences, recognising that meaning is constructed through individuals’ interactions with their personal and professional contexts [14]. It emphasises that interpretation is not neutral but is influenced by the dynamic relationship between the researcher and the participant, as well as the broader social context in which the experiences are situated. Hermeneutic phenomenology was selected for its capacity to illuminate how participants may construct meaning around IP through their relational experiences and career trajectories [13, 15].

### Participants

Healthcare simulation educators who scored 60 or higher on the Clance Impostor Phenomenon Scale (CIPS) in a previous study, indicating frequent to intense IP experiences based on Clance’s categorisation, were invited for interview. In the earlier study, 148 simulation educators from nine countries completed an online survey [1]. The results showed that 46.6% (*n* = 69) of participants experienced elevated levels of IP. For this current study, these 69 educators were invited to participate in semi-structured interviews. Using criterion sampling, we invited these educators to participate in interviews. Demographic variables including years of experience, gender, and experience working in different contexts within simulation education were monitored to provide context for understanding participants’ experiences.

### Data collection

Semi-structured interviews were the primary method of data collection since they allow for the elicitation of rich, thick descriptions, essential for capturing the complexity of participants’ narratives [16].

The interviews focused on broad areas of inquiry, encouraging participants to reflect on key moments and feelings surrounding IP in their professional lives. These areas included the following:

- Early career experiences and the development of identity as a health professional or educator.
- Impostor feelings in different simulation roles.
- Coping strategies and the impact of IP on well-being and career development.
- The role of support networks in managing impostor feelings.

The interviewer (K. F.) initiated each interview using open-ended questions, allowing participants to shape the conversation. Exploratory questions were used to delve deeper into certain areas, but the interviews remained largely participant-led, reflecting our commitment to uncovering their lived realities. We focused on achieving data sufficiency, gathering enough data to develop nuanced interpretations while remaining open to new insights throughout the process [17]. This fluid approach also allowed us to remain open to shifting themes, in line with hermeneutic inquiry, where understanding unfolds through interaction and interpretation. Interviews were conducted via video conference, lasting between 40 and 60 min, and were audio-recorded.

We avoided providing an operational definition of IP to participants, aiming instead to understand how participants made sense of this concept. Although participants had previously completed the CIPS, their scores were only shared midway through the interview when the researcher (K. F.) asked if they would like to know their score. This approach was taken to ensure that participants’ reflections on IP were not influenced or constrained by their CIPS score, allowing for a more open and authentic exploration of their experiences.

### Data analysis

Following each interview, the audio-recordings were transcribed using NVivo Transcription software and checked for accuracy (K. F.). This process enabled the researcher (K. F.) to be fully immersed in the data, reflecting on both what participants said and how and what they might have meant [18, 19].

Transcripts were read holistically, with simultaneous listening to the audio-recordings, during which the researcher took notes to reflect on the essence of each participant’s experience. This note-taking process deepened the connection with participants’ narratives and allowed for rich insights into their lived experiences. Line-by-line analysis followed, identifying phrases central to the experience of IP (K. F.). Key phrases were coded in NVivo to capture recurring concepts. Following the hermeneutic circle approach, analysis involved a dynamic movement between specific experiences shared by individuals and the broader understanding of the phenomenon, allowing for deeper interpretation of meaning [20]. This iterative process involved returning to earlier interviews as new insights were identified, ensuring that our interpretations remained grounded in participants’ narratives while capturing both individual and collective experiences [15].

K. F. analysed interviews as they were conducted, identifying candidate subthemes from each transcript, with this initial analysis informing subsequent interviews. Over several months, these initial interpretations were shared with S. C., who independently reviewed selected transcripts and K. F.’s preliminary codes. K. F. and S. C. then critically examined and refined the preliminary coding to develop a thematic structure that captured the depth and complexity of participants’ lived experiences.

**Fig. 1:**
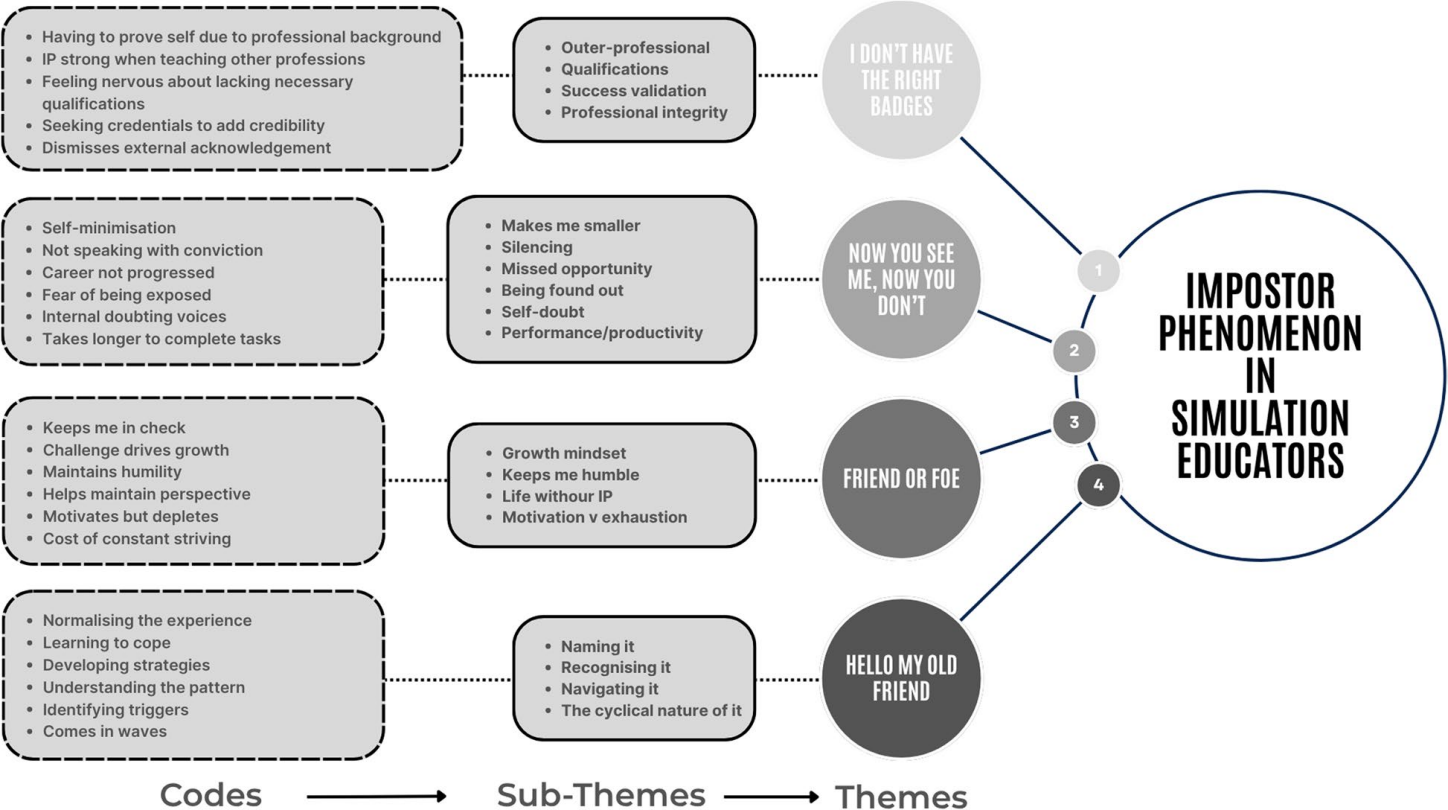
Thematic map of IP analysis

An illustration of how themes were generated from verbatim quotes is provided in Table 1. The analytical progression from initial codes through subthemes to the four themes is demonstrated in Fig. 1.

**Table 1:** Illustration of theme generation

Participant quotes	Initial codes	Subthemes	Major themes
“[CHSE], just got filed away, that little bit of paper. Even after getting the badges still doesn't feel…enough.” (Quinn)	Even after getting the badges still doesn’t feel enough	Qualifications	I don’t have the right badges
“I always worry that somebody is going to find out that I'm not as good as they might think I am” (Gina)	Fear of being discovered	Being found out	Now you see me, now you don’t
“On my good days, I think it keeps me grounded. It stops me from getting too confident and pushes me to keep improving. But on my bad days, it's exhausting. It makes me feel like I'll never be good enough no matter how hard I try” (Fran)	Both good and bad	Motivation vs exhaustion	Friend or for
“Every time I start something new, I'm hit with that same wave of doubt—like, this time they'll figure out I don't belong. But after a while, I settle in and realize I can do it. It's just a pattern that I've had to learn to manage” (Elizabeth)	Recurring pattern of doubt with new challenges	Recognising it	Hello my old friend

### Reflexivity

As healthcare simulation educators and researchers, we acknowledged our potential influence on interpretations. The primary researcher (K. F.), an experienced simulation educator who has experienced IP herself, maintained a personal reflexive journal throughout the study. This journalling supported critical self-reflection, documenting immediate reactions to interviews and examining potential influences of her own experiences as a simulation educator on data interpretation. This practice supported the hermeneutic circle approach, moving between individual experiences and collective understanding. Regular team debriefing sessions with co-authors, who brought perspectives from health professions education (S. C.), educational psychology (S. H.), and international simulation and health professions education (D. N.), enabled critical examination of assumptions and interpretations. Through this iterative process, we returned to earlier interviews as new insights were identified, ensuring interpretations remained grounded in participants’ narratives. S. C. independently reviewed selected transcripts and preliminary coding, strengthening credibility while maintaining sensitivity to participants’ lived experiences.

### Ethical considerations

Ethical approval for the study was obtained from The University of Western Australia Human Research Ethics Committee (RA/4/20/5061). All participants provided written informed consent prior to their involvement, ensuring they were fully aware of the study’s aims and their rights. To protect participant confidentiality, pseudonyms were assigned based on the alphabet, generating names for each participant from A to T. This allowed for a coherent narrative in reporting the findings while maintaining participants’ anonymity. Any identifying information was removed from transcripts. Additionally, to further protect participants’ privacy, geographical regions were used instead of specific countries during the reporting process. Given the relatively small size of the professional healthcare simulation, this approach minimised identification of participants, preserving their privacy.

## Findings and discussion

Our findings are integrated with discussion, an approach

that aligns with phenomenology methodology where interpretation is inherently part of the descriptive process [21]. This integration enhances the interpretive depth and narrative flow of the research.

In total, over 900 min of interview data were collected. Table 2 summarises the demographic characteristics of the participants, ranked according to their score on the CIPS. Table 3 outlines the four themes we identified, which encapsulate the participants’ experiences of IP in healthcare simulation education.

**Table 2:** Participant characteristics

Pseudonym	CIPS score	Age	Healthcare discipline	Years in sim	Geographical region
Fran	91	39	Medicine	8	Oceania
Mary	91	49	Nursing	16	North America
Jenny	90	46	Paramedicine	8	North America
Gina	86	58	Nursing	16	Oceania
Donna	85	51	Nursing	18	Europe
Iris	84	52	Nursing	6	North America
Natalie	84	48	Nursing	12	Europe
Helen	83	34	Other professional background	5	North America
Leanne	81	50	Pharmacy	8	North America
Tamara	80	41	Other professional background	13	North America
Rebecca	77	30	Physiotherapy	3	North America
Poppy	74	31	Nursing	7	Oceania
Oscar	71	38	Medicine	9	Oceania
Kate	70	42	Nursing	5	Europe
Quinn	65	39	Physiotherapy	4	North America
Adam	63	31	Other professional background	8	North America
Ben	62	42	Medicine	8	Oceania
Claire	61	48	Other professional background	19	North America
Elizabeth	61	57	Nursing	14	North America
Stacey	61	47	Other professional background	17	North America

^*^Participants have been ordered from higher to lower scores of IP

**Table 3:** Key themes and descriptions of IP experiences

Theme	Description
*I don’t have the right badges*	Participants frequently compared themselves to peers, feeling they lacked the qualifications or validation to be successful. Many described a need to prove their worth by metaphorically collecting “badges”
*Now you see me, now you don’t*	IP often led participants to minimise their presence in professional settings. This “shrinking” behaviour resulted in missed opportunities and a diminished sense of identity as a simulation educator
*Friend or foe*	Participants had a contradictory relationship with IP. While it motivated some to strive for professional growth, for others, it was a source of insecurity that hindered their performance
*Hello my old friend*	Participants described the cyclical nature of IP, where feelings of self-doubt resurfaced with each new professional challenge, despite having previously experienced similar experiences

These themes encapsulate how participants’ narratives reflect ongoing negotiation, tension, and personal growth, contributing to the growing body of research that frames IP as a socially constructed phenomenon rather than a pathological condition.

## Theme 1: I don’t have the right badges — seeking validation in a world valuing badges

This theme captures the educators’ pervasive sense of not being qualified or accomplished enough compared to their peers. We described four subthemes: ‘outer professional’ (feeling like an outsider among other healthcare professionals), ‘qualifications’ (pursuing formal credentials), ‘success validation’ (seeking external acknowledgment), and ‘professional integrity’ (concerns about being worthy of one’s position).

The metaphor of ‘badges’ draws directly from participants’ experiences, as Donna explained: ‘We're always chasing letters… we need to get the badge a bit like, if you like, an analogy of a girl guide you get your badge for everything. I think nursing is a bit like being a girl guide, you've got your sash and your badges’. This constant pursuit of credentials stemmed from a perceived need to prove legitimacy, as she further reflected: ‘And the fact I'm doing an EduD I'm still chasing the letters because I feel if I don't have a doctorate, then I'm not genuine’.

The ‘outer-professional’ experience was powerfully illustrated by Adam: ‘I was the only person at my level in the college of Medicine that didn't have a higher degree, that didn't have a master's or a doctorate. I was doing I was being asked to do a lot of work that faculty normally would be the ones to do at my previous institution. And I was being asked to sit on a lot of committees and be part of a lot of conversations with a bunch of people that had MDs and PhDs and EdDs and I think I was very, very nervous about that’.

This hierarchical pressure was deeply embedded in healthcare simulation education. Donna observed: ‘I think very sadly, in the simulation world, it's very hierarchical still… they get doctor, even though it's essentially a bachelor's degree in medicine and surgery. So, they get the honorary title, and the term “doctor” brings a set of assumptions’. This dynamic influenced how educators approached their professional development. Helen explained: ‘But I think it's mostly to get the credential. I feel like it's entirely to, like, backup my job titles and stuff with a credential. Which is probably a little bit silly, because I don't think I would get any more money for it because I have a Master's and a PhD’. The impact of these hierarchical structures was felt daily. Helen further reflected: ‘But I think it's also, you know, there's one of those, like, few little things that occasionally get under your skin, and it's that, you know, all of their assistants, and a lot of our residents and people, like, they're all doctor, and I'm Helen’.

Concerns about ‘professional integrity’ persisted even when others offered validation. Claire said: ‘Because when you hear people say like, oh, yeah, like you're great. Like, it means nothing when you have those internal conversations. It's hard to drown out those doubting voices’.

This relentless pursuit of validation through credentials aligns with Chakraverty’s observation that professionals, regardless of career stage, often feel compelled to chase external markers of success [22, 23]. However, as participants shared, these accomplishments provided only temporary relief from impostor feelings, perpetuating a sense of ‘not enough’. The transient nature of this relief suggests that external validation alone cannot resolve the deeper uncertainties that characterise IP.

Ibarra suggests that professional identity is continuously formed through experimentation with new roles, which can exacerbate the sense of never fully arriving [24]. This theoretical perspective helps explain why simulation educators, who frequently transition between different roles and responsibilities, might be particularly vulnerable to IP. Healthcare simulation institutions and professional societies, with their emphasis on qualifications and measurable outcomes, can magnify these impostor feelings by reinforcing a culture that equates competence with visible markers of success.

The paradox participants described, where badges both signal belonging and deepen self-doubt, manifested in their daily professional lives. Donna reflected: ‘I almost feel I have to then give it like the world's best CV of myself just to get that credibility. But my medical colleagues never do that, it's just accepted’. This experience exemplifies how the very credentials meant to establish legitimacy often reinforced feelings of difference and inadequacy. Such insights underscore the need to reconsider how achievement is recognised in simulation education.

## Theme 2: Now you see me, now you don’t: the art of shrinking

This theme captures how participants’ impostor feelings led them to minimise their presence in professional settings. This ‘shrinking’ behaviour manifested as a carefully choreographed dance of being present yet invisible — a complex interplay between internal experience and external presentation.

Adam described how IP led him to ‘take up less space’ in professional settings. He reflected on moments where he deliberately withdrew from discussions to avoid being noticed, even though he had valuable contributions to make: ‘I don’t want to put myself out there, in case they realise I don’t belong’. This strategic withdrawal aligns with Leary et al.’s findings that individuals experiencing IP often engage in protective self-presentation behaviours, deliberately minimising their visibility as a way to manage professional interactions and protect against potential negative evaluation [25]. While such behaviour may offer immediate psychological protection, participants described how this self-imposed invisibility ultimately resulted in lost opportunities and a diminished sense of identity as a simulation educator.

Jenny’s experience further illustrated how impostor feelings affected the ability to speak up: ‘I’ve gotten quieter in meetings. I used to contribute more, but now I hold back. I don’t know if it’s because I’m afraid of being wrong or because I feel like what I have to say isn’t important. Either way, I’ve stopped speaking up as much’*.* This self-minimisation eroded confidence over time, creating a self-perpetuating cycle of silence.

The anxiety surrounding the possibility of failure or being exposed as inadequate created a cycle of silence. Fran shared how this dynamic played out: ‘I constantly second-guess myself, and in doing so, I miss out on opportunities. It’s easier to stay quiet than to risk being wrong or looking foolish’*.* These acts of self-minimization represent calculated efforts to avoid the emotional toll of failure or judgment.

This pattern of professional invisibility is particularly significant in simulation education, where educators must continuously transition between roles as teacher, facilitator, and learner. Such role flexibility should ideally foster confidence and adaptability. Instead, for those experiencing IP, these transitions can become moments where the art of shrinking is most precisely executed, with educators carefully calibrating their visibility to avoid exposure. As this practiced silence builds, it corrodes not only individual confidence but also the collective knowledge sharing that is vital to the simulation education community.

The carefully crafted practice of withdrawal described by participants reflects a complex interplay between personal doubt and professional expectations. In healthcare simulation, where educators are often expected to demonstrate expertise across multiple domains, this artful shrinking becomes a refined survival strategy, preventing individuals from fully inhabiting their professional identities. The cumulative effect extends beyond individual career trajectories, potentially limiting innovation and collaboration within the field.

## Theme 3: Friend or foe? The dual nature of IP

Participants demonstrated a complex and sometimes contradictory relationship with IP. As a ‘friend’, it kept them humble and drove them to strive for improvement. However, as a ‘foe’, it served as a persistent source of insecurity and anxiety.

They spoke about how IP pushed them to work harder, preventing them from becoming complacent. However, this drive for perfection was also seen as detrimental to their well-being.

Claire’s reflection captured this ambivalence: ‘On my good days, I think it keeps me grounded. It stops me from getting too confident and pushes me to keep improving. But on my bad days, it’s exhausting. It makes me feel like I’ll never be good enough no matter how hard I try’. Her experience echoes Rosenscruggs et al.’s findings about the potential for reframing IP as a positive force, a way of maintaining curiosity and resisting complacency [26]. However, Claire’s account also demonstrates the emotional cost of this constant striving.

Some participants had developed sophisticated strategies for managing this duality. Poppy described her conscious effort to reframe impostor feelings as a catalyst for growth: ‘I don’t let IP hold me back anymore. Instead, I use it to remind myself that I always have more to learn. It’s a motivator, but it’s also draining because it never lets you rest’. Her approach to IP highlights how some educators harness these feelings productively.

Conversely, Fran expressed frustration at how IP prevented her from fully enjoying her successes: ‘It’s like I can never celebrate the wins because I’m always thinking about the next thing I need to prove. It’s exhausting. I don’t want to live my whole career chasing validation’. Her experience aligns with Thomas and Bigatti’s findings, which link perfectionism with the emotional exhaustion that accompanies IP [27].

The line between healthy motivation and emotional exhaustion proved remarkably thin. For many participants, the energy spent proving oneself came at a significant cost, leading to burnout, missed opportunities, and compromised well-being. This delicate balancing act was particularly challenging in simulation education, where educators must constantly demonstrate competence across multiple domains while maintaining the emotional capacity to support learners effectively.

What we saw in these narratives was a nuanced understanding of IP not as a uniformly negative force but as a complex phenomenon that requires careful management. This duality aligns with Rohrmann et al.’s findings that IP represents a dysfunctional personality style with both protective and impairing aspects [28]. While some educators found ways to channel their impostor feelings into professional development, others struggled with the relentless pressure to prove their worth. This variation in experience parallels what Neureiter and Traut-Mattausch describe as the complex interplay between IP and career-relevant motivational constructs, where impostor feelings can simultaneously drive achievement and inhibit career development [29].

Such findings suggest that institutional support and professional development programs need to acknowledge both the potential benefits and risks of IP. Rohrmann et al. argue that effective interventions must recognise IP’s dual nature rather than treating it as purely problematic [28]. Supporting educators requires developing sustainable strategies that harness IP’s motivational aspects while mitigating its potential for professional burnout.

## Theme 4: Hello my old friend — the cyclical nature of IP

The cyclical nature of IP was a common experience among participants, with many noting that these feelings would resurface in their careers, especially when faced with new challenges. Despite their accomplishments, participants described how each new job or project reignited insecurities.

Elizabeth provided a vivid description of this recurring cycle: ‘Every time I start something new, I’m hit with that same wave of doubt—like, this time they’ll figure out I don’t belong. But after a while, I settle in and realise I can do it. It’s just a pattern that I’ve had to learn to manage’*.* Her experience aligns with Ajjawi and Higgs’ observation that impostor feelings are particularly triggered by transitions and new responsibilities [19]. For Elizabeth, recognising the cycle allowed her to manage through periods of doubt more effectively.

Not all participants found such equilibrium. Fran, on the other hand, reported that her impostor feelings had intensified with time: ‘It’s gotten worse in the last few years. I’ve taken on a more senior role, and the expectations are so high. I feel like I’m under constant pressure to prove myself, and I don’t know how to manage it anymore’. Her experience highlights how increased responsibility can exacerbate impostor feelings, even for those with substantial professional experience.

Adam spoke about how impostor feelings were triggered whenever he mentored others: ‘Whenever I mentor someone, those impostor feelings creep back in. I start wondering if I’m really qualified to give advice, even though I know I’ve been doing this for years’*.* His account shows how even seasoned professionals are not immune to these cycles of self-doubt, particularly when taking on roles that require them to assert their expertise.

These recurring cycles challenge the traditional view of IP as primarily an individual psychological phenomenon requiring personal coping strategies. While earlier literature emphasised individual interventions [2], our findings align with more recent theoretical developments suggesting that IP operates at both individual and institutional levels [30]. Downing et al. argue that IP constitutes a dysfunctional personality style that requires more than just individual coping strategies it calls for systemic support and community engagement [30]. This is relevant in healthcare simulation education, where educators frequently transition between different roles and responsibilities, each transition potentially triggering a new cycle of impostor feelings.

The persistence of IP throughout participants’ careers, even at high level of achievement, suggests that rather than viewing IP as a temporary phase to be ‘overcome’, it might be more productive to conceptualise it as what Downing et al. describe as an ongoing process requiring continuous management through both individual and institutional responses [30]. This reconceptualisation has important implications for how organisations approach leadership development and support.

Through these four interconnected themes, we see how IP shapes the professional lives of healthcare simulation educators. From the persistent pursuit of validation through ‘badges’ to the artful practice of shrinking from opportunity, the double-edged nature of IP as both motivator and barrier, and its cyclical resurfacing throughout careers, our findings show that IP is deeply embedded in professional identity formation.

Rather than a temporary challenge to be overcome, IP is a complex phenomenon requiring ongoing management through both individual and institutional responses. For simulation educators, whose roles demand flexibility, expertise across multiple domains, and constant adaptation to new challenges, the impact of IP is particularly significant. These insights suggest the need for a fundamental shift in how we understand and address IP within healthcare simulation education — moving beyond individual coping strategies to create environments that acknowledge, support, and nurture professional growth at all career stages.

### Implications for the healthcare simulation community

Given the prevalence of IP among simulation educators and its impact on wellbeing and career development, there is a need to consider how professional communities and institutions might better support educators experiencing these feelings. Future research could explore the role of mentorship programmes, communities of practice, and institutional support systems in helping simulation educators manage IP. Professional societies should also reconsider how credentials and certifications (‘badges’) are positioned within the community, shifting emphasis from markers of legitimacy to milestones in ongoing professional development. Professional development programs should go beyond technical skills, however, offering spaces for educators to reflect on their experiences and explore strategies for managing IP. As Rosenscruggs et al. note, mentorship programmes that foster authentic conversations about IP can reduce isolation and create opportunities for educators to develop professional confidence [26].

The dynamic nature of simulation education, where educators shift between roles, necessitates a community-focused approach. This approach should support professional identity formation, offering educators opportunities to experiment with roles and build confidence over time. Ibarra emphasises the importance of identity experimentation in professional adaptation, suggesting that simulation educators need both freedom and support to fully inhabit their professional identities [24].

### Strengths and considerations

This study makes several important contributions. First, it provides novel insights into the lived experience of IP among simulation educators, a previously understudied population. This qualitative study, underpinned by hermeneutic phenomenology, provides deep understanding of the phenomenon’s impact on educators. While participants were purposively sampled based on their CIPS scores from a previous quantitative study, the current research focused on exploring their lived experiences through in-depth interviews. Additionally, the international sample provides broad perspective on this experience across different contexts.

Several aspects of the study warrant consideration. The participants who chose to share their experiences were those who were willing to discuss their experiences of IP. This self-selection may mean that we have omitted the lived experience of those unwilling or unable to discuss their experiences.

The in-depth interpretive interviews provided rich insights into how simulation educators experience and make meaning of IP. As an experienced simulation educator, the interviewer (K. F.) shared her own vulnerabilities with IP which may have created a space for participants to share their own vulnerable experiences. While this insider perspective enhanced the depth of our interpretations, we acknowledge that participants may have shaped their narratives based on perceived social expectations within the simulation education community. The complex and personal nature of IP means that some aspects of the experience may remain difficult to articulate, even in thoughtful dialogue.

Another important consideration concerns the cultural context of the participants. As the study focused exclusively on English-speaking educators, the findings may not capture how IP is experienced across diverse cultural and linguistic contexts. Cultural variations in perceptions of professionalism, hierarchy, and success are likely to shape how IP is experienced. For instance, in cultures with different social norms around failure or self-promotion, the manifestation of IP might differ significantly from what was observed in this study.

Finally, although the study sheds light on the cyclical nature of IP, the data represents participants’ experiences at specific points in time. Longitudinal research could explore how IP evolves across careers and in response to new challenges, offering a more dynamic understanding of the phenomenon.

### Future research directions

Future research could explore several key areas. First, while our study primarily focused on English-speaking educators, exploring how cultural contexts influence the experience of IP would provide a more comprehensive understanding of how IP manifests globally. Second, longitudinal studies could examine how IP evolves throughout educators’ careers, particularly during key transitions and role changes. Third, deeper exploration of how mentorship programs and communities of practice shape educators’ experiences of IP could inform approaches to supporting professional growth and identity development. Finally, research examining how organisational culture influences IP experiences could inform institutional approaches to supporting simulation educators.

## Conclusion

This study offers an in-depth exploration of the persistent and cyclical nature of IP among healthcare simulation educators, revealing the complex ways in which these feelings intersect with professional identity, relationships, and career trajectories. Participants shared narratives of self-doubt, anxiety, and fear of failure, yet they also described moments where IP became a motivator, driving self-improvement. These dualities highlight that IP is not purely negative but a dynamic phenomenon, capable of fostering both personal growth and professional exhaustion.

Our findings suggest that while impostor feelings can serve as catalysts for excellence, they often come at a significant emotional cost, leading to reduced well-being and missed opportunities for professional development. Addressing IP requires more than individual coping strategies. It calls for systemic interventions within the healthcare simulation community, including mentorship, professional development programmes, and communities of practice, to foster environments where educators feel safe to share their experiences openly.

Given the prevalence of IP and its impact on wellbeing and career development, there is a need for the simulation community to acknowledge and address these experiences. Starting conversations about IP is an important first step in supporting educators who may be experiencing these feelings.

The healthcare simulation community, with its established practices of debriefing and reflection, is well-positioned to support educators through intentional dialogue, reflective practice, and institutional support. By acknowledging the complex nature of IP and offering sustained support, we can empower educators to thrive, not despite their impostor feelings but through embracing and managing them.

## Data Availability

Our human research ethics approval (RA/4/20/5061) does not permit entire transcripts to be shared beyond the research team. However, excerpts of the de-identified transcripts are available on request.

## Abbreviations

IP: Impostor phenomenon
CIPS: Clance Impostor Phenomenon Scale
